# AMPK Phosphorylates LMX1b to Regulate a Brainstem Neurogenic Network Important for Control of Breathing in Neonatal Mice

**DOI:** 10.3390/ijms26010213

**Published:** 2024-12-30

**Authors:** Traci L. Marin, Christopher G. Wilson, Miguel Lopez Ramirez, Wei Sun, Atul Malhotra, Brendan Gongol

**Affiliations:** 1 Department of Respiratory Therapy, Victor Valley College, Victorville, CA 92395, USA; 2 Department of Medicine, University of California San Diego, San Diego, CA 92093, USA; 3 Department of Basic Sciences, Division of Physiology, Loma Linda University, Loma Linda, CA 92354, USA; 4 Department of Pharmacology, University of California San Diego, San Diego, CA 92093, USA; 5 VA San Deigo Medical Center, San Diego, CA 92161, USA; 6 Institute for Integrative Genome Biology, University of California, Riverside, CA 92697, USA

**Keywords:** AMPK, Lmx1b, ventilatory drive, respiratory center maturation, apnea, brainstem, apnea of prematurity, AOP, sudden infant death syndrome, SIDS

## Abstract

Ventilatory drive is modulated by a variety of neurochemical inputs that converge on spatially oriented clusters of cells within the brainstem. This regulation is required to maintain energy homeostasis and is essential to sustain life across all mammalian organisms. Therefore, the anatomical orientation of these cellular clusters during development must have a defined mechanistic basis with redundant genomic variants. Failure to completely develop these features causes several conditions including apnea of prematurity (AOP) and sudden infant death syndrome (SIDS). AOP is associated with many adverse outcomes including increased risk of interventricular hemorrhage. However, there are no pharmacological interventions that reduce SIDS and AOP prevalence by promoting brainstem development. AMP-activated protein kinase (AMPK) is a kinase that regulates ventilatory control to maintain homeostasis. This study identifies a signaling axis in which the pharmacological activation of AMPK in vivo via metformin in brainstem ventilatory control centers results in the phosphorylation of LIM homeobox transcription factor 1-beta (Lmx1b), a key player in dorsal–ventral patterning during fetal development. The phosphorylation of Lmx1b transactivates a neurogenic interactome important for the development and regulation of ventilatory control centers. These findings highlight the potential for metformin in the treatment and prevention of AOP.

## 1. Introduction

Approximately 15% percent of all pregnancies end in premature delivery affecting roughly 13 million infants annually [1]. Although pre-term infants are viable after 23 weeks’ gestation, incomplete development of cardiopulmonary anatomical features, particularly the development of the central brainstem ventilatory control centers, can result in apnea of prematurity (AOP) and periodic breathing. This inability to regulate ventilatory drive results in increased mortality in premature infants. AOP is a developmental disorder attributed to a lack of cellular differentiation and the maturation of central brainstem cardio-ventilatory control centers resulting in stochastic breathing patterns that consist of cessation of breathing for >20 s accompanied by bradycardia, cyanosis, or pallor [2]. The incidence of AOP in neonates is inversely related to gestational age and birth weight, with almost 50% of infants born less than 32 weeks’ gestation experiencing a degree of AOP [3]. This finding suggests that incomplete development of the central pattern generator for breathing is a primary contributing factor. The ventilatory control centers are intricately connected and comprise spatially oriented clusters of neurons and glia, including the retrotrapezoid nucleus, lateral reticular nucleus, Bötzinger complex, pre-Bötzinger complex, and caudal ventral respiratory group, which make up an important anatomical architecture enabling the integration of neural- mechano-, and chemo-afferents that regulate ventilation and maintain normal arterial blood gas values [4].

Current interventional modalities for AOP, such as tactile or mechano-stimulation, pharmacological stimulants (methylxanthines), and positive pressure ventilation, fail to promote ventilatory control center development and reduce the duration of AOP. Further, untreated AOP requiring the use of high levels of supplemental oxygen or mechanical ventilation can result in severe comorbidities including bronchopulmonary dysplasia, retinopathy of prematurity, interventricular hemorrhage, and mortality [3,5]. Despite the high prevalence of AOP and the understanding that developmental modulation of atonal bHLH transcription factor 1 (Atoh1), paired-like homeobox 2b (Phox2b), and developing brain homeobox 1 (Dbx1) [6] are important for ventilatory control center development essential for survival across mammalian species, the molecular mechanisms that regulate ventilatory control center complex spatial positioning and maturation are unresolved.

AMP-activated protein kinase (AMPK), a heterotrimeric kinase consisting of an α, β and γ subunit, is activated by stimulations that increase the AMP/ATP ratio and plays a central role in re-establishing energy balance at the cellular, tissue, and organismal levels. At the cellular level, AMPK increases aerobic respiration by increasing mitochondrial biogenesis and function [7]. At the tissue level, AMPK regulates arterial dilation, which increases perfusion to meet local cellular O_2_ metabolic demand. At the organismal level, AMPK regulates ventilatory control, which facilitates ventilation and oxygenation at the alveolar–capillary membrane [8]. Although the role of AMPK in energy metabolism is well established, the scope of AMPK’s role across a variety of cellular functions, such as cellular differentiation and development, is limited. Evidence of AMPK double α1 and α2 knockout embryonic lethality while maintaining mouse embryonic fibroblast cellular viability suggests AMPK plays an important role in development and cellular differentiation [9]. However, the scope of AMPK’s role in the development of respiratory control centers is currently unknown.

Metformin is an AMPK agonist and a common drug used to treat diabetes mellitus that can cross both the blood–brain and placental barriers [10,11]. Neonatal administration of metformin reduces the incidence of AOP in murine animals and reduces the oxidative lung tissue damage that occurs with O_2_ delivery in neonates [12,13]. Importantly, metformin can be safely administered to pregnant women with polycystic ovarian syndrome, which significantly increases the live birth rate [14,15]. However, the scope of prenatal medications that reduce the incidence of AOP is limited, and the mechanistic basis for metformin-decreased infant mortality is unknown.

The aim of this study is to explore the activation of AMPK via metformin as an important mechanism for ventilatory control center development by activating LIM homeobox transcription factor 1-beta (Lmx1b), a regulator of dorsal–ventral patterning during fetal development [16]. Given that dorsal–ventral patterning is important for the anatomical placement of ventilatory control centers during development, and neuron differentiation is required for the control of ventilation, this study explores important roles of AMPK in the prevention and treatment of AOP. The translational applications of this study explore the mechanistic potential for the pharmacological intervention with the AMPK agonist, metformin, to reduce the incidence and co-morbidities associated with AOP, such as interventricular hemorrhage.

## 2. Results

### 2.1. AMPK Regulates the Control of Ventilation That Can Be Rescued via Metformin Administration

**Figure 1:**
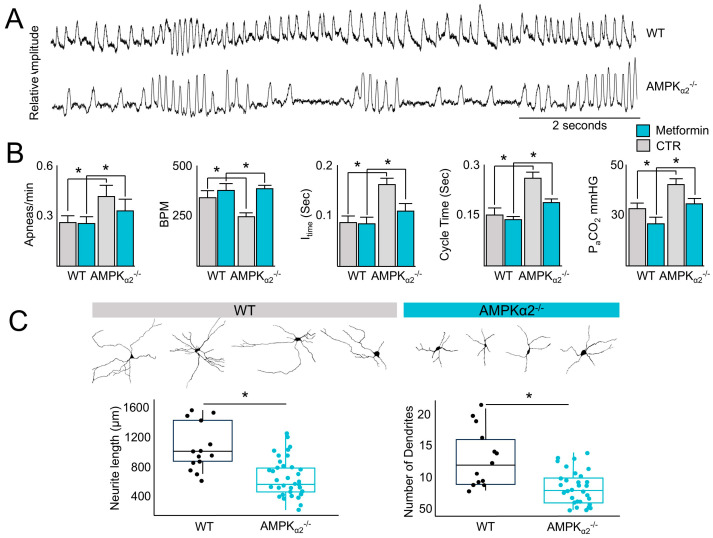
AMPK governs ventilatory patterns and respiratory control center neurite outgrowth. (**A**) Plethysmography recording demonstrating irregular ventilatory patterns in AMPKα_2_^−/−^ newborn mice compared with WT littermates. Upward deflections above baseline represent a single breath. (**B**) Plethysmography recordings were quantified with or without metformin treatment for the number of apneas/min, the number of breaths/min (BPM), the inspiratory time (I_time_), and the cycle time demonstrating metformin (200 mg/kg) rescued ventilatory aberrancies in AMPKα_2_^−/−^ newborn mice. Following plethysmography, PaCO_2_ was measured. (**C**) Images of brainstem sections at the level of the preBötzinger complex with neurons visualized using Golgi–Cox stain (left panels), representative neurons (upper panels), and quantification of neurite outgrowth showing impaired growth in AMPKα_2_^−/−^ newborn mice compared with WT littermates. Results are from 3 independent experiments. * Indicates *p* < 0.05.

The cardiopulmonary system is an essential organ system that maintains cellular function in the distal tissues, including the maintenance of aerobic metabolism, and the regulation of ventilatory drive, which are vital components of this system. Given the important role of AMPK in the regulation of energy metabolism and the central role of the control of ventilation to meet metabolic demand, whether AMPK regulates the control of ventilatory drive was tested. Baseline plethysmography performed on newly born mice revealed an increase in stochastic breathing patterns with prolonged periods of apnea in *AMPKα_2_*^−/−^ (Supplementary Figure S1) but not wild-type (WT) mice (Figure 1A), with an increase in the number of apneas, a lower breath rate, longer inspiratory and cycle times, and higher partial pressure of arterial carbon dioxide (PaCO_2_) (Figure 1B). However, treatment with metformin (Sigma-Aldrich, St. Louis, USA, 200 mg/kg, intraperitoneal), a known activator of AMPK, rescued the bradypnea and hypercapnia in *AMPKα_2_^−/−^ mice* (Figure 1B), suggesting a compensatory role for AMPKα_1_ [8,17]. Since *AMPKα_2_^−/−^ mice* are hypercapnic, the possibility that AMPK plays an important role in neuron function in ventilatory control centers was investigated by measuring the length of neurite outgrowth from tissue sections obtained from AMPKα_2_^−/−^ and WT littermates. Compared with WT littermates, neurite outgrowths in ventilatory control centers were shorter in AMPKα_2_^−/−^ mice (Figure 1C). Taken together, these data suggest that AMPK plays an important role in controlling ventilatory drive.

### 2.2. AMPK Induces a Neurogenic Regulatory Network

**Figure 2:**
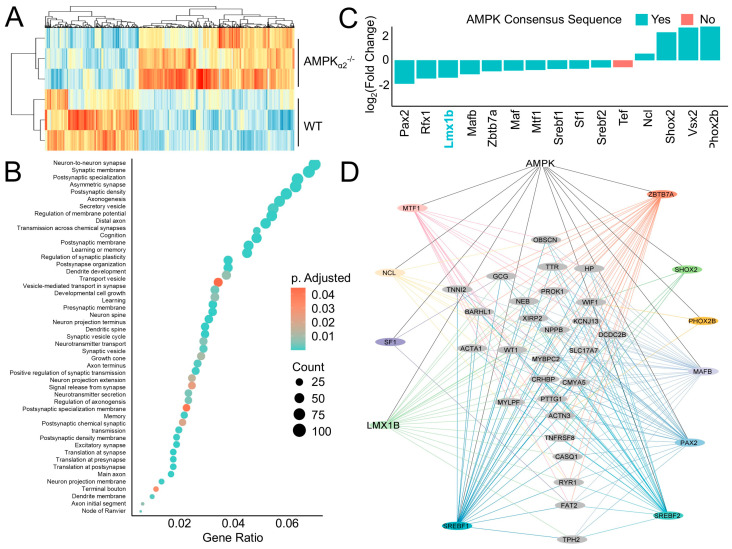
AMPK regulates a transcriptional network related to ventilatory control center development. (**A**) Heatmap of DEG expression levels from RNA-seq results generated from brainstem samples harvested from AMPKα_2_^−/−^ and WT littermates. (**B**) Functional enrichment analyses of DEGs identified in the AMPKα_2_^−/−^ compared with WT RNA-seq data. (**C**) Fold changes of transcription factors expressed in brainstem samples. Blue indicates presence of an AMPK phosphorylation consensus sequence. (**D**) AMPK interactome constructed by mapping AMPK phosphorylation sites to transcription factors that are expressed in brainstem regions and mapping their consensus sequences to the promoter regions +/− 1000 base pairs from the transcriptional start site. Differentially expressed genes (DEGs) are represented as grey nodes, transcription factors are colored nodes in the periphery of the graph with their color-coded consensus sequence connections to DEG promoters indicated by the lines. Abbreviations: LIM homeobox transcription factor 1-beta (Lmx1b), MAF bZIP transcription factor (Maf), MAF bZIP transcription factor B (Mafb), metal response element binding transcription factor 1 (Mtf1), nucleolin (Ncl), paired box 2 (Pax2), paired-like homeobox 2b (Phox2b), regulatory factor X1 (Rfx1), SHOX homeobox 2 (Shox2), steroidogenic factor 1 (Sf1), sterol regulatory element binding transcription factor 1 and 2 (Srebf1, Srebf2), thyrotroph embryonic factor (Tef) visual system homeobox 2 (Vsx2), and zinc finger and BTB domain containing 7A (Zbtb7a).

AMPK influences ventilatory patterns by regulating neuronal activity in brainstem ventilatory centers [8], yet the mechanistic basis of this phenotype is unknown. To address this, RNA sequencing (RNA-seq) was performed on brainstem ventilatory control center tissue samples from AMPKα_2_^−/−^ mice and their WT littermates. Differential expression analyses comparing AMPKα_2_^−/−^ with WT littermate samples identified differentially expressed genes (DEG) that separate into two distinct clusters by genotype (Figure 2A). Functional enrichment analyses using DEGs identified pathways related to neuron development including synapatogenesis, signaling and plasticity, axonogenesis, and dendrite formation (Figure 2B). To explore the mechanistic basis of these DEGs, the RNAseq dataset was filtered to identify differentially expressed transcription factors that were then cross-referenced with a database of putative AMPK targets leading to the identification of 10 transcription factors that are both expressed in the brainstem RNA-seq data and are putative AMPK phosphorylation targets (Figure 2C) [18]. Of these transcription factors, Lmx1b is involved in dorsal–ventral patterning; paired box 2 (Pax2) is important for early central nervous system development [19]; nucleolin (Ncl), MAF bZIP transcription factor (Maf) and MAF bZIP transcription factor B (Mafb) are involved in neuronal development and maturation [20,21]; regulatory factor X1 (Rfx1) is essential for embryonic development [22]; sterol regulatory element binding transcription factor 1 and 2 (Srebf1, Srebf2) regulate embryonic dopaminergic neuronal development [23]; visual system homeobox 2 (Vsx2) is important neuronal function [24]; Phox2b regulates neuron maturation and ventilatory pattern [6]; zinc finger and BTB domain containing 7A (Zbtb7a) is a neuronal epigenetic regulator [25]; SHOX homeobox 2 (Shox2) regulates neuronal signaling and ion channel function [26]; metal response element binding transcription factor 1 (Mtf1) protects against oxidative stress in neurons [27]; steroidogenic factor 1 (Sf1) is involved in many aspects of neuronal signaling and energy homeostasis [28]; and thyrotroph embryonic factor (Tef) is important circadian rhythm regulation [29].

The transcriptional targets of these transcription factors were explored by identifying transcription factor binding sites in the promoters of DEGs. Then, a consensus-sequence-derived AMPK regulatory network was constructed depicting the putative direct regulatory influence AMPK has on gene transcription (Figure 2D). Taken together, these results indicate AMPK plays an important role in ventilatory control and they identify several AMPK-regulated transcription factors, including Lmx1b, that are involved in neuronal development and signaling.

### 2.3. AMPK Induces LMX1B

**Figure 3:**
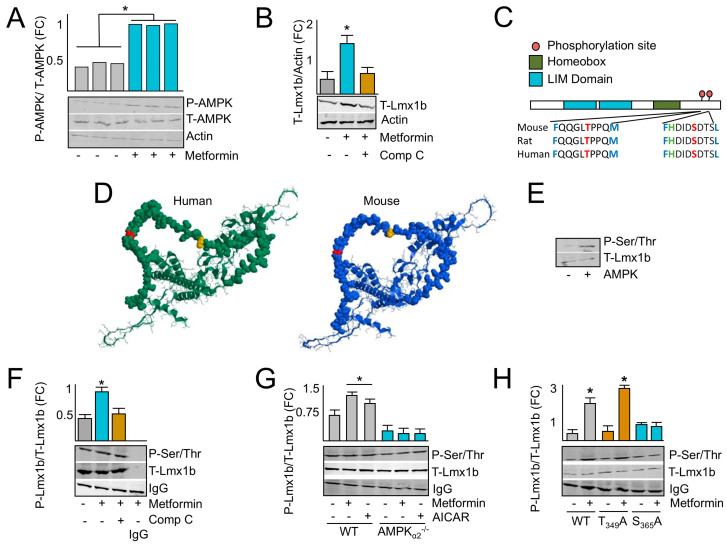
AMPK upregulates and phosphorylates Lmx1b on serine-365. (**A**) Immunoblotting from lysates harvested from neurons treated with metformin for 10 min to demonstrate AMPK activation via phosphorylation. (**B**) Immunoblotting from cell lysates showing metformin increased Lmx1b expression but was inhibited by co-administration of Comp. C. (**C**) Illustration of the domains of LMX1B, the location of the phosphorylation sites, and the flanking residues that meet the minimum AMPK phosphorylation consensus sequence. (**D**) α-fold Lmx1b structural prediction with the location of T349 (orange) and S365 (red). (**E**) Kinase assay indicating phosphorylation of Lmx1b in reactions containing AMPK but not in reactions without AMPK. (**F**) Immunoblot analysis of immunoprecipitated Lmx1b from WT neuron lysates showing increased phospho-Lmx1b in cells treated with metformin but not when co-treated with Comp. C. (**G**) Lmx1b immunoprecipitation followed by immunoblotting showing increased phospho-Lmx1b in neurons isolated from WT mice when treated with metformin or AICAR, but not in neurons isolated from AMPKα_2_^−/−^ mice. (**H**) Lmx1b immunoprecipitation followed by immunoblotting from neurons isolated from WT mice, transfected with WT, T349A or S365A constructs and then treated with metformin, or left untreated, demonstrating that metformin increased phosphorylation of S365 but not T349. Bar colors in bar plots distinguish sample groups. Results are from 3 independent experiments. * Indicates *p* < 0.05.

To explore the molecular underpinning of AMPK’s role in the brainstem, neurons isolated from the brainstem regions of AMPKα_2_^−/−^ and WT mice were treated with AMPK agonists 5-aminoimidazole-4-carboxamide ribonucleotide (AICAR) and metformin. Metformin activated AMPK within 10 min as demonstrated by the AMPK phosphorylation of T-172 on the α subunit, a marker of AMPK activation (Figure 3A). Since AMPK is activated by metformin in isolated neurons, we explored how AMPK transcriptionally regulates neuron development through the regulation of Lmx1B. Lmx1b was selected for several reasons. First, it is differentially down-regulated in the RNA-seq analysis that was performed comparing the brainstems of AMPK^−/−^ to AMPK^+/+^ mice indicating that AMPK activation plays an important role in its induction and downstream signaling effects (Figure 2C). Second, it is a predicted AMPK target which suggests that the phosphorylation of Lmx1b may regulate its downstream signaling activity (Figure 2C,D). Finally, Lmx1b is important for dorsal–ventral positioning of anatomical features during development, which may be key for the development of the structure–function relationship and spatial positioning of ventilatory control centers within the brainstem. To test this, we treated neurons isolated from WT brainstems with metformin and measured Lmx1B protein expression levels. As expected, metformin increased Lmx1b expression, which was inhibited by the AMPK inhibitor compound C (Comp. C) (Figure 3B). Taken together, these results indicate that AMPK induces the expression of Lmx1b, and that the expression level of Lmx1b can be induced pharmacologically with AMPK agonists.

### 2.4. AMPK Phosphorylates LMX1B at Serine 365

AMPK consensus sequence searching of the proteome identified two putative AMPK phosphorylation sites on Lmx1b, threonine (T) 349, and serine (S) 365, located in the C terminus of the protein that are conserved across at least 43 species (Figure 3C). To investigate the accessibility of the phosphorylation site, the structure of Lmx1b was predicted with α fold, and the locations of T348 and S365 were identified (Figure 3D). These predicted structures indicate that both T348 and S365 are located on a peptide chain at the surface of the protein that is accessible to phosphorylation. To validate if AMPK can phosphorylate Lmx1b, kinase assays were performed using recombinant AMPK and Lmx1b, which illustrated an increase in the Lmx1b phosphorylation signal (P-Lmx1b) in reactions containing AMPK (Figure 3E). Normalization of Lmx1b levels before immunoprecipitation of Lmx1b followed by immunoblotting for phospho-serine/threonine from cells treated with metformin with or without Comp. C for 10 min indicated an increase in P-Lmx1b with metformin treatment but not when cells were co-treated with Comp. C (Figure 3F). Next, neurons isolated from the brainstem regions of AMPKα_2_^−/−^ and WT mice were treated with AMPK agonists AICAR or metformin for 10 min. Then, Lmx1b protein levels were normalized between samples, Lmx1b was immunoprecipitated, and immunoblotting with a phospho-serine/threonine antibody was performed (Figure 3G). Treatment with both metformin and AICAR increased the level of P-Lmx1b but not as significantly in AMPKα_2_^−/−^ cells. To determine the specificity of the phosphorylation site, cells were transfected with native, T349A, or S365A Lmx1b constructs and then treated with or without metformin. Immunoprecipitation followed by immunoblot analysis indicated an increase in native and T349A Lmx1b phosphorylation following metformin treatment, but not when left untreated or if S365A Lmx1b was expressed (Figure 3H). Taken together, these results indicate that AMPK phosphorylates Lmx1b at S365.

### 2.5. Lmx1b Governs a Transcriptional Network Important for Ventilatory Center Neuron Maturation

**Figure 4:**
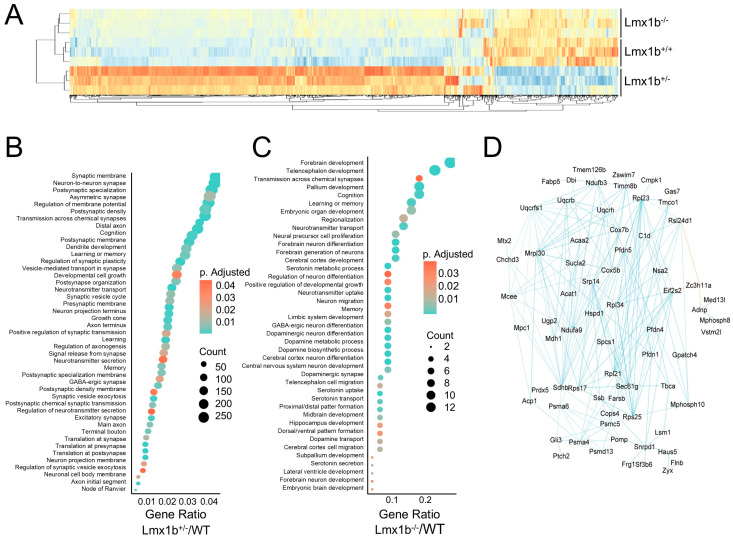
LMX1B activated a transcriptional profile of genes involved in the neuronal development in the brainstem. (**A**) Heatmap and cluster analysis of the expression levels of the union of DEGs identified in RNA-seq differential expression analyses comparing tissue samples collected from the brainstems of Lmx1b^−/−^ and Lmx1b^+/−^ mice compared to Lmx1b^+/+^ mice. Over representation pathway analysis of DEGs identified in the Lmx1b^+/−^ compared to WT mice (**B**) and Lmx1b^−/−^ compared to WT mice (**C**) RNA-seq data. (**D**) Interactome illustrating the relationships between genes identified with promoter sequences flanking +/− 1000 bp of the transcriptional start site.

Lmx1b is an important transcription factor that regulates dorsal–ventral patterning during development. However, its role in the development of ventilatory centers essential for the control of ventilation has not been explored. Although Lmx1b^−/−^ is embryonic lethal, RNA sequencing was performed on embryonic brainstem ventilatory centers harvested from prenatal WT, Lmx1b^+/−^, and Lmx1b^−/−^ mice to elucidate the influence Lmx1b has on the ventilatory centers and identify genes involved in ventilatory center patterning during development. Differential expression analyses comparing Lmx1b^−/−^ or Lmx1b^+/−^ with WT littermates identified DEGs that cluster the samples by genotype (Figure 4A). Functional enrichment analyses identified several dysregulated pathways from the Lmx1b^+/−^ samples related to neuronal development including synaptogenesis, signaling and plasticity, axonogenesis, and dendrite formation (Figure 4B). Further, the dysregulated pathways found in the Lmx1b^−/−^ samples included those involved in central nervous system (CNS) anatomical development, neuronal development, and neuronal signaling, such as forebrain development, telencephalon development, transmission across chemical synapses, regionalization, neural precursor cell proliferation, and regulation of neuron differentiation (Figure 4C). Next, genes were identified that have Lmx1b binding motifs flanking +/− 1000 base pairs from the transcription start site and visualized these results as an AMPK-Lmx1b signaling interactome (Figure 4D). Many of the interactome genes are involved in cell cycle activity and proliferation, underscoring their role in development and neurogenesis. For example, cytochrome c oxidase complex subunit 5B (Cox5b) and eukaryotic translation initiation factor 2 subunit beta (Eif2s2) are important for cell proliferation and growth [30,31]; prefoldin subunit 5 (Pfdn5) is necessary for protein folding, migration and proliferation [32]; ribosomal proteins such as ribosomal proteins L21 and L34 regulate cell cycle via protein synthesis and DNA replication [33]; and signal recognition particle 14 (Srp14) is involved in cell cycle and cell adhesion [34].

### 2.6. Metformin Administration Induces Fetal Expression and Phosphorylation of Lmx1b

**Figure 5:**
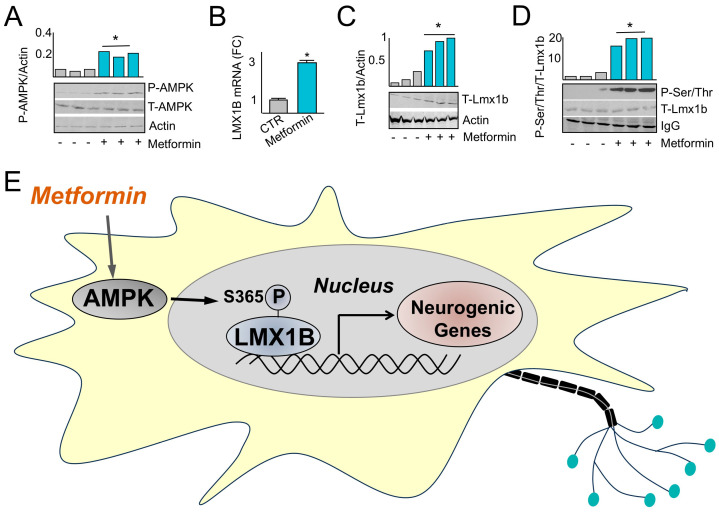
AMPK increases Lmx1b expression and phosphorylation. (**A**) Immunoblotting indicating increased phosphorylation of AMPK Thr-172 in fetal brainstem samples obtained from dams treated with metformin (200 mg/kg). qPCR analysis (**B**) and immunoblotting (**C**) using lysates demonstrating that metformin increases Lmx1b expression in fetal brainstem samples obtained from dams treated with metformin. (**D**) Immunoprecipitation followed by immunoblotting showed an increase in Lmx1b phosphorylation in fetal brainstem samples obtained from dams treated with metformin (200 mg/kg). (**E**) Graphical abstract illustrating that metformin-activated AMPK increases Lmx1b phosphorylation, which in turn transcriptionally activates neurogenic genes. * Indicates *p* < 0.05.

The administration of pharmacological agents to mothers at risk of premature delivery to promote the development of ventilatory control centers may reduce the incidence of AOP, the need for interventional approaches, and sequelae including interventricular hemorrhage. Therefore, whether the AMPK-Lmx1b ventilatory control signaling axis can be pharmacologically activated in utero via administration of metformin was explored. Pregnant dams were administered metformin following 5 days of gestation for 10 days prior to harvesting brainstem fetal tissue. Brainstem fetal tissue harvested from dams administered metformin illustrated an increase in the level of AMPK phosphorylation on Thr-172 (Figure 5A) and increased total Lmx1b mRNA (Figure 5B) and protein (Figure 5C) levels. In addition, readjusting sample loading to the level of T-Lmx1b followed by T-Lmx1b immunoprecipitation and immunoblotting with phospho-serine/threonine antibody indicated increased P-Lmx1b levels in samples obtained from dams administered metformin compared with those that were not (Figure 5D). These results indicate that pharmacological administration of metformin to pregnant mothers activated the AMPK-Lmx1b signaling axis. In summary, the results of this study demonstrate that metformin activates AMPK, which phosphorylates Lmx1b at S365 to transactivate genes important for neuronal development and signaling (Figure 5E).

## 3. Discussion

This study explores the mechanistic basis for AMPK regulation of ventilatory control in the CNS and identifies an AMPK-Lmx1b signaling network that mediates neuronal development, maturation, signaling, and homeostasis in ventilatory control centers. These findings identify important insights into the functions of AMPK for ventilatory center development that are likely to have profound clinical applications for the treatment and prevention of developmental breathing disorders and sequelae including AOP, sudden infant death syndrome (SIDS), or neonatal interventricular hemorrhage [35].

AMPK contains two isoforms of the catalytic α subunit (α_1_, α_2_) that are both highly conserved across evolution and demonstrate independent and overlapping functions, including the regulation of growth factors that promote brain development [36,37]. Data illustrated in Figure 1 indicated that AMPKα_2_^−/−^ elicits a stochastic breathing pattern, yet the AMPK agonist, metformin, can return the respiratory rate to wild type levels. These data are supported by studies indicating the α_1_ subunit is an important regulator of ventilatory control and suggest that functional redundancies exist between the catalytic subunits for the maintenance of ventilatory control [8,38], a common finding among genes regulating phenotypes essential for organismal life [39].

Phenotypically, many of the pathways identified in the AMPKα_2_^−/−^ compared to WT mice were involved in cellular proliferation or other aspects of neuron development and neurite outgrowth (Figure 2D). However, studies on AMPK’s role in neuronal function have mixed results underscoring potential brain-region-specific or development-specific AMPK-regulated outcomes [40,41,42,43]. In addition, while these pathways have been primarily studied in association with neurological pathologies, they are likely an essential component of development and a reflection of micro-environmental changes that occur in the brainstem during maturation via the activation of the AMPK-Lmx1b signaling axis.

Clinically, AMPK can be activated by AICAR, metformin, phenformin, 2-deoxy-D-glucose (2DG), salicylate, telmisartan, and A-769662 [44]. Of these AMPK agonists, metformin is unique because it has a low risk of side effects, crosses both the blood–brain barrier and placental barrier, and can be administered to pregnant mothers with low risk of fetal side effects [10,11]. Human development requires dorsal–ventral asymmetry, which is regulated by Lmx1b throughout fetal development in the CNS and developing limb bud [16]. However, the molecular underpinnings by which Lmx1b orchestrates this dorsal–ventral asymmetry are still unclear. In vivo studies demonstrate that Lmx1b^−/−^ is embryonic-lethal, and developing mice lack limb-bud dorsalization and develop only ventral surfaces [45]. Within the CNS, Lmx1b is required for the development of serotonin-secreting neurons and the differentiation and migration of neurons within the dorsal spinal cord [46]. However, the effects of Lmx1b on the development of the ventilatory control centers have not been determined. Although the spatial organization of Lmx1b and its involvement with dorsal–ventral patterning in the brainstem is still unknown, data illustrated in Figure 3 and Figure 5 indicate that AMPK activation increases Lmx1b expression that subsequently regulates a transcriptional network controlling pathways involved in neuron development. This signaling network may facilitate the anatomical distribution of neuronal centers during development and may reduce the incidence and duration of AOP in the neonatal population (Figure 2B).

The AMPK phosphorylation site on L”x1b ’s located in a predicted unstructured region of the protein outside of the LIM domains that consists of two contiguous zinc fingers that mediate protein–protein interactions and the regulation of transcription [47]. In addition, the phosphorylation sites are C-terminal to a homeobox domain that is involved in DNA binding and the regulation of anatomical feature development [48]. Although it is possible that S365 phosphorylation results in Lmx1b conformational changes that change the overall flexibility of the flanking peptide chain, there are very few predicted structural insights that suggest how the phosphorylation of S365 mediates Lmx1b biological functions. One possibility is that S365 phosphorylation is involved in mediating protein–protein interactions with co-regulatory transcription factors that influence gene expression and ventilatory center development. Nonetheless, the translational relevance of this signaling pathway is supported by data illustrated in Figure 5 indicating that pharmacological intervention with metformin activates the AMPK-Lmx1b signaling axis in utero, which may accelerate brainstem development in pregnancies at risk for pre-term delivery for the prevention of AOP.

Although the experiments and analyses performed throughout the results of this manuscript provide foundational evidence for the association between AMPK, Lmx1b, and the potential pharmacological effects of metformin on AOP, we recognize several limitations of this study. Data illustrated in Figure 5 indicated only correlational evidence supporting the AMPK-Lmx1b signaling axis in vivo. In addition, there is a lack of AMPK α_1_ and α_2_ brainstem-specific, double-knockout mice that are important for understanding isoform specificity and genomic redundancy. Further, spatial sequencing data to decipher the cell-type-specific effects, as well as the effect the AMPK-Lmx1b signaling axis, have on the spatial–temporal development of ventilatory control centers are lacking. Finally, there are a lack of human data to support the translational application of metformin in ventilatory center development and its effects on AOP in the neonatal population. All of these studies are important for demonstrating the translational relevance of this study, particularly since there is a lack of pharmacological interventions that address AOP. Clinically, current therapies for pregnant mothers at risk for pre-term delivery include administration of steroids to promote pulmonary and neurological development [49], which has several side effects including hyperglycemia, bone density loss, and impaired immune response [50]. Post-delivery interventions include the administration of caffeine or positive pressure ventilation to pre-term neonates, which fail to address the developmental immaturity in the brainstem and result in a variety of cardiopulmonary co-morbidities, including interventricular hemorrhage, a common cause of fetal mortality [51]. The administration of AMPK agonists, such as metformin, may provide a new approach for the prevention and management of AOP and other disorders related to brainstem immaturity.

## 4. Materials and Methods

Ethics: All procedures were performed in accordance with NIH guidelines. Animal studies were approved by the Loma Linda Institutional Animal Care and Use committee (IACUC protocol #819004) on 21 July 2015.

Transfection: Lmx1b constructs were purchased from custom DNA constructs LLC., Islandia, NY, USA (https://customdnaconstructs.com/home-2/ accessed on 8 August 2018). Transfection was conducted with lipofectamine LTX (Cat # 15338100) using cells at 70% confluence according to the manufacturer’s instructions using 1 μg of respective constructs.

Immunoblotting: Cells were lyzed in 10 mM tris pH = 7.4, 0.1 M NaCl, 1 mM EDTA, 1mM EGTA, 1mM NaF, 20 mM Na_4_P_2_O_7_, 2 mM Na_3_VO_4_, 0.1% SDS, 0.5% sodium deoxycholate, 1% Triton X-100, 10% glycerol, 1mM phenylmethylsulfonyl fluoride, and protease inhibitors. Resulting cell lysates were resolved using SDS-polyacrylamide gel electrophoresis (SDS-PAGE) and transferred to polyvinylidene fluoride (PVDF) membranes (VWR Cat# 27376-991). Following transfer, PVDF membranes were blocked for 1 h in blocking buffer [5% milk in Tris buffered saline with Tween 20 (TBST) (50 mM Tris-HCl, pH 7.4, 150 mM NaCl, 0.1% Tween 20)]. Membranes were then washed three times with TBST and incubated with primary antibody diluted in blocking buffer overnight. Next, membranes were incubated with a secondary antibody that was diluted in blocking buffer. After washing three times in TBST, membranes were incubated with chemiluminescent horseradish peroxidase (HRP) substrate, and immunoblots were developed. The antibodies used in this manuscript are as follows: Phosphoserine/threonine (Invitrogen Cat# MA5-38234, Waltham, MA, USA), AMPK P-172 (ThermoFisher Scientific Cat# 44-1150G, Waltham, MA, USA), T-AMPK (Santa Cruz Cat# sc-74461), T-Lmx1b (Invitrogen Cat# PA5-78395), and Actin (Santa Cruz Cat# sc-47778, Dallas, TX, USA).

RT-qPCR: After decanting the medium, cells were immediately lyzed using TRIzol reagent from Life Technologies (cat # 10296-028, Carlsbad, CA, USA). RNA was subsequently purified according to the manufacturer’s instructions. After isolation, RNA was converted to cDNA using Promega reverse transcriptase according to the manufacturer’s instructions (cat # M1701). qPCR was then performed using SYBR green qPCR master mix (Bio-Rad, Thane, India) in a Bio-Rad CFX96 thermocycler cycling between 94 °C, 58 °C, and 72 °C. Relative mRNA abundance was calculated using the Δ-Δ ct method with the following primer sets: Lmx1b Forward: CGGGATCGGAAACTGTACTG, LMX1B Reverse: AGCAGAAACAGCCCAAGTG, Gapdh Forward: AGGCCGGTGCTGAGTATGTC, and Gapdh reverse: TGCCTGCTTCACCACCTTCT.

Kinase assays: AMPK kinase assays were performed with a buffer containing 50 mM 2-[4-(2-hydroxyethyl) piperazin-1-yl] ethanesulfonic acid (HEPES), 0.375 mM AMP, 0.375 mM ATP, 9 mM MgCl_2_, and 2 mg of target proteins with or without 11 pM AMPK (Sigma Aldrich cat # A1733-10UG, St. Louis, MO, USA). Reactions were incubated at 37 °C for 1 h. Following incubation, proteins were resolved by SDS-PAGE, stained with Coomassie blue, and immunoblotting was performed.

RNA-Sequencing: Tissue samples were homogenized in TRIzol reagent from Life Technologies (cat # 10296-028) and RNA was isolated according to the manufacturer’s instructions. RNA was prepared for sequencing using the NEXTflex™ Rapid Directional qRNA-Seq™ Library Prep Kit (Bioo Scientific, Austin, TX, USA) and sequenced using an Illumina HiSeq 2000.

Bioinformatics analyses. Bioinformatics analyses were performed in *R* programming language version 4.4.2 with support from the *Bioconductor* and *CRAN* packages. RNA-seq analyses were performed using the *systempipeR Bioconductor* package version 3.20. Briefly, reads were aligned using hisat2. Following read alignment, differential expression analyses were performed with *edgeR version 3.20*, and cluster analyses were performed along with the generation of heatmaps with the *pheatmap* package version 3.20. Binding site analyses were performed by pattern-matching consensus sequences obtained from the *JASPAR* database (https://jaspar.elixir.no/ accessed on 10 January 2018) to genomic sequences obtained from *Ensembl*. Functional enrichment analyses were performed using the *clusterprofiler* package version 3.20. Protein–protein interactions were predicted with string, and network interactomes were created with *Cytoscape* version 3.10.3.

Statistical Analysis. Parametric data were analyzed using student’s *t*-test or ANOVA. An unpaired *t* test with Welch’s correction was used if the variance was not equal across groups. For, non-parametric data, the Mann–Whitney U test with exact method was used to analyze differences between groups. *p* < 0.05 was considered statistically significant.

Neuron Imaging: Brainstem tissue was isolated prior to perfusion with phosphate buffered saline via cardiac puncture to remove any blood. Once isolated, brain sections were washed with 0.9% saline and 4% paraformaldehyde (PFA), embedded in optimal cutting temperature compound (OCT) (fisher Scientific Cat# 23-730-571), frozen at −80 °C, sectioned into 100–200 um sections using a microtome, and fixed into glass slides. Tissue sections were stained with Golgi–Cox stain as previously described [52,53]. Briefly, brain sections were immersed in Golgi–Cox solution (50 mL of potassium dichromate solution, 50 mL of the mercuric chloride solution, 40 mL of the potassium chromate solution, and 100 mL of dd-H_2_O) and protected from light for 24 h. The Goli–Cox solution was then changed and samples were stored, protected from light, for an additional 7 days. Next, samples were submerged in protectant solution (300 g sucrose, 10 g polyvinylpyrrolidone, and 300 mL ethylene glycol dissolved in 500 mL phosphate buffered saline) and stored for 24 h at 4 °C. Protectant solution was then changed and samples were stored in fresh protectant solution for an additional 7 days at 4 °C. Samples were then washed three times with phosphate buffered saline prior to imaging. Z-stacked images of neurons were obtained using a confocal microscope, and Sholl analysis was performed to measure dendrite length and the number of dendrites emanating from each neuron cell body using *Image J* version 16.0.

Plethysmography: Plethysmography was performed as previously described [54,55]. The breathing pattern was assessed using non-invasive, whole-body, awake plethysmography (custom chambers, *LabChart* software, version 7.38, AD Instruments). Plethysmography experiments were performed at the same time of day to avoid diurnal cycling. All mice were acclimated to the plethysmography chamber for 30 min before beginning experiments. The latter 15 min of this time were used for baseline recordings. Mice were removed from the chamber between trials and allowed to feed with dams in their nests for at least one hour between plethysmography trials. Metformin was prepared and injected (10 mg/kg) intraperitoneally (i.p.) in sterile saline (~200 μL depending on the mouse), and mice were then placed in the chamber and recorded for 30 min. The chamber was heated to 32 °C and bias flow (200 mL/min) was humidified room air (21% O_2_). All experiments were conducted in a quiet, dimly lit room. Flow, tidal volume, minute ventilation, inspiratory (Ti) and expiratory (Te) times, interbreath intervals, and baseline fitting/detection to quantify breathing parameters score apneas and respiratory pauses were recorded. Shannon and Sample entropy, measures of signal complexity/variability, were calculated. Plethysmography data were acquired immediately before anesthetizing the animal for tissue harvest. Using these methods, we are able to assess the awake breathing pattern as well as the anesthetized breathing pattern and central drive in the animals. All breathing patterns (slow, fast, sighing, and gasping rhythms) were quantified and analyzed by our software, freely available at our GitHub repository (https://github.com/drcgw/BASS accessed on 10 June 2023).

Neuron Isolation: Neurons were isolated by dissecting brainstem regions into 2 mm sections on a petri dish containing ice cold Hank’s balanced salt solution (HBSS) purchased from Thermo Fisher Scientific. Dissected sections were titrated in a 15 mL conical tube containing 8 mL Neurobasal Medium purchased from Thermo Fisher Scientific using a 1 mL pipette. Next, cells were filtered through a 70 µm mesh filter and then plated into Poly-D-Lysine coated coverslips. Cells were housed in a 5% CO_2_ incubator environment at 37 °C and neurobasal medium was changed roughly every third day.

Animal Care and Pharmacological Intervention: Mice were kept on a 12/12 light cycle in the Loma Linda Animal Care Facility (ACF). Mice aged postnatal days 1 to 5 were used. At the conclusion of the experiment, mice were placed in an anesthesia induction chamber, deeply anesthetized at 4% isoflurane in 100% O_2_, and transcardially perfused with chilled saline and 4% paraformaldehyde (PFA). The brain was removed, placed in 4% paraformaldehyde for 24 h, changed to 30% sucrose in PFA and OCT, and stored at −80 °C until ready for sectioning (see above).

## 5. Conclusions

This study demonstrates that AMPK plays a role in the development of ventilatory control centers in the CNS via an AMPK-Lmx1b signaling network. These findings identify functions of AMPK for respiratory center development that are likely to have profound clinical applications for the treatment of AOP, and may extend to the prevention of sudden infant death syndrome (SIDS), or other comorbidities related to ventilatory control center development [35].

## Data Availability

All data from these experiments will be available after publication by request from the authors. RNA-seq data are available on Gene Expression Omnibus (GEO) under the accession number GSE284418.

## Supplementary Materials

The following supporting information can be downloaded at: https://www.mdpi.com/article/10.3390/ijms26010213/s1.
