# Supplementary material for: AMPK Phosphorylates LMX1b to Regulate a Brainstem Neurogenic Network Important for Control of Breathing in Neonatal Mice
Source: Int J Mol Sci. 2024 Dec 30;26(1):213. doi: 10.3390/ijms26010213 (PMC11720625; doi:10.3390/ijms26010213)
Supplement: Supplementary file 1 [file ijms-26-00213-s001.zip › ijms-3386347-supplementary.pdf]

Supplementary Materials:

A

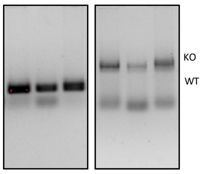

Figure S1. Genotyping. (A) Genotyping PCR reactions indicating lower AMPK$\alpha_2^{-/-}$ mice in the right three lanes and WT mice in the left three lanes
